# Interplay of Nrf2 and BACH1 in inducing ferroportin expression and enhancing resistance of human macrophages towards ferroptosis

**DOI:** 10.1038/s41420-022-01117-y

**Published:** 2022-07-19

**Authors:** Dmitry Namgaladze, Dominik C. Fuhrmann, Bernhard Brüne

**Affiliations:** 1 Institute of Biochemistry I, Faculty of Medicine, Goethe-University Frankfurt, Frankfurt, Germany; 2 Fraunhofer Institute for Translational Medicine and Pharmacology ITMP, Frankfurt, Germany; 3 German Cancer Consortium (DKTK), Partner Site Frankfurt, Frankfurt, Germany; 4 Frankfurt Cancer Institute, Goethe-University Frankfurt, Frankfurt, Germany

**Keywords:** Cell death, Immune cell death

## Abstract

Compared to cancer cells, macrophages are inert to lipid peroxidation-triggered, iron-dependent cell death known as ferroptosis. Mechanisms underlying macrophage resistance towards ferroptosis are largely obscure. Here, we show that human primary macrophages respond to RSL3, a ferroptosis-inducing inhibitor of glutathione peroxidase 4, by upregulating mRNA expression of the iron transporter ferroportin. RSL3 induces lipid peroxidation, and both, lipid peroxidation as well as ferroportin induction were attenuated by liproxstatin-1, an inhibitor of lipid peroxidation and ferroptosis blocker. At the same time, system x_c_^–^ inhibitor erastin fails to elicit lipid peroxidation or ferroportin expression. Ferroportin induction in response to RSL3 demands nuclear accumulation of the redox-sensitive transcription factor Nrf2 and downregulation of the transcriptional repressor BACH1. Silencing ferroportin or Nrf2 increases the cellular labile iron pool and lipid peroxidation, thereby sensitizing cells towards ferroptosis following RSL3 treatments. In contrast, silencing BACH1 decreases the labile iron pool and lipid peroxidation, enhancing macrophage resistance towards ferroptosis. Our findings reveal Nrf2, BACH1, and ferroportin as important regulators, protecting human macrophages against ferroptosis.

## Introduction

Ferroptosis is a recently discovered mode of cell death driven by lipid peroxidation [1, 2]. Initially described in the context of anti-tumor therapy [3], ferroptosis is now recognized to play important roles in the pathophysiology of disease, such as ischemia-reperfusion, neurodegenerative diseases, or acute kidney injury [2, 4]. Ferroptosis also affects various immune processes. It is critical for the homeostasis of follicular helper T cells [5], drives neutropenia during systemic lupus erythematosus [6], and mediates the demise of tumor-infiltrating CD8^+^ T cells in response to fatty acids in the tumor microenvironment [7]. In contrast to cells of the adaptive immune system, innate immune cells, in particular, macrophages are relatively inert to ferroptosis [8]. The mechanisms underlying macrophage resistance against ferroptosis remain poorly understood.

Three parameters largely define sensitivity to ferroptosis in a given cell: the amount of polyunsaturated fatty acids in membrane lipids, the balance of lipid reactive oxygen species (ROS)—generating and scavenging systems, and the availability of free ferrous iron [1]. Ferrous iron is known to readily promote lipid peroxidation through Fenton chemistry. Thus, its cellular levels are tightly controlled through uptake, storage, and export mechanisms [9]. Iron export is mostly facilitated by the membrane transporter ferroportin (gene name SLC40A1) [10]. Ferroportin is highly expressed in duodenal epithelial cells, where it mediates the export of nutritionally derived iron into systemic circulation [11], as well as in macrophages phagocytizing red blood cells [12]. The amount of ferroportin in the plasma membrane is regulated at multiple levels. Transcriptional activation of ferroportin mRNA expression is achieved through the redox-sensitive transcription factor nuclear factor erythroid 2-related factor 2 (Nrf2, gene name NFE2L2) [13, 14], whereas a transcriptional repressor BTB and CNC homology 1 (BACH1) suppresses ferroportin expression [13]. In addition, translational regulation of ferroportin is mediated by iron-responsive proteins [15]. Finally, the hepatically produced small protein hepcidin downregulates cell surface ferroportin levels by inducing its endocytosis and degradation [16].

Human macrophages can be sensitized towards ferroptosis by depletion of the iron storing ferritins [17]. Whether additional protective mechanisms operate in response to ferroptosis induction in human macrophages is unknown. Here, we show that ferroportin mRNA expression is elevated in human primary macrophages treated with the ferroptosis-inducing glutathione peroxidase 4 (GPX4) inhibitor RSL3. This involves Nrf2 stabilization and BACH1 downregulation in response to RSL3. Silencing ferroportin and Nrf2 aggravates ferroptosis, whereas silencing BACH1 is cytoprotective.

## Results

**Fig. 1:**
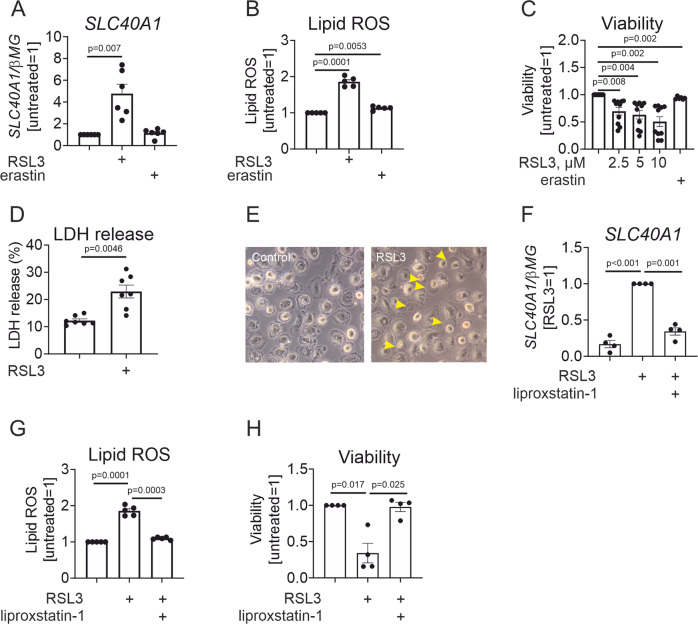
RSL3 induces ferroportin gene expression and ferroptosis in human macrophages. **A** mRNA expression of SLC40A1 in MΦ exposed to 10 µM RSL3 for 24 h. **B** Lipid ROS levels in MΦ treated with 10 µM RSL3 or 10 µM erastin for 6 h. **C** Viability of MΦ exposed to indicated concentrations of RSL3 or 10 µM erastin for 24 h. **D**, **E** LDH release and morphology of MΦ treated with 10 µM RSL3 for 24 h. Arrowheads indicate membrane blistering. **F**–**H** SLC40A1 mRNA expression (**F**), lipid ROS (**G**), and viability (**H**) of MΦ treated with 10 µM RSL3 in the presence or absence of 250 nM liproxstatin for 6 h (**G**) or 24 h (**F**, **H**). Data are presented as means ± SEM (*N* ≥ 4).

Initially, we evaluated the impact of ferroptosis-inducing stimuli on ferroportin mRNA expression in primary human macrophages (MΦ). Treatment of MΦ with the GPX4 inhibitor RSL3, but not system x_c_^–^ inhibitor erastin, increased ferroportin (SLC40A1) mRNA (Fig. 1A) expression. Unfortunately, we could not reliably detect human ferroportin protein using commercially available antibodies in our system. Next, we measured lipid peroxidation after treatments with RSL3 and erastin by flow cytometry using a lipid ROS-specific fluorescent sensor BODIPY C11 581/591. As seen in Fig. 1B, the lack of lipid ROS production in erastin-treated MΦ correlated with the failure of erastin to elicit ferroportin expression. At the same time, RSL3 induced 2-fold increases of lipid ROS after 6 h incubation. Corroborating these data, only RSL3 caused a concentration-dependent loss of macrophage viability measured by the CellTiter-Blue® assay (Fig. 1C), whereas erastin induced less than a 10% decrease in viability. Cytotoxicity induced by RSL3 was independently confirmed using a LDH release assay (Fig. 1D). Morphologically, dying MΦ showed plasma membrane blistering, which is characteristic of ferroptotic cells [18] (Fig. 1E). To address the role of lipid peroxidation in ferroportin induction by RSL3, we employed a potent lipid peroxidation inhibitor and ferroptosis blocker liproxstatin-1 [19]. Treating the MΦ with RSL3 in the presence of liproxstatin-1 abolished ferroportin mRNA induction (Fig. 1F), lipid ROS generation (Fig. 1G), and preserved MΦ viability (Fig. 1H).

**Fig. 2:**
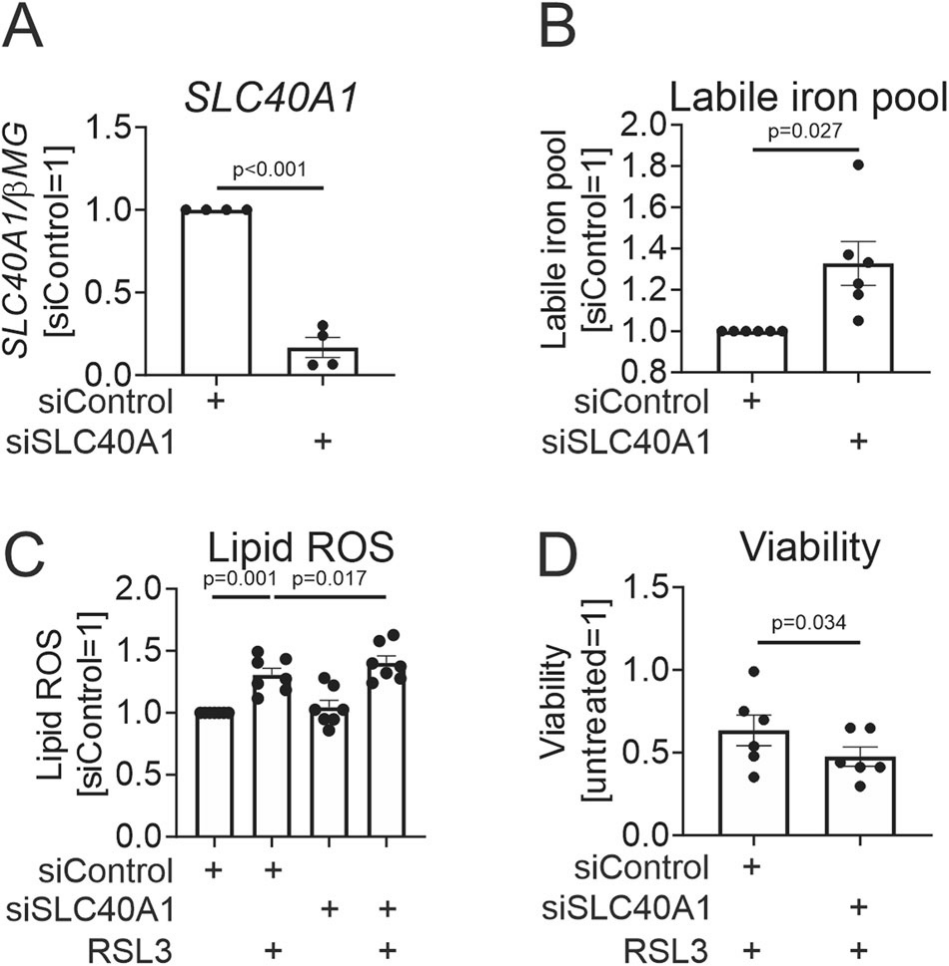
Ferroportin silencing increases the labile iron pool and exacerbates RSL3-triggered lipid ROS and ferroptosis in human macrophages. **A**, **B** mRNA expression of SLC40A1 (**A**), and LIP measurements (**B**) in MΦ transfected with control or SLC40A1 siRNAs for 96 h. **C**, **D** Lipid ROS (**C**) and viability (**D**) of MΦ transfected with control or SLC40A1 siRNAs for 96 h prior to treatments with 10 µM RSL3 for 6 (**C**) or 24 h (**D**). Data are presented as means ± SEM (*N* ≥ 4).

Next, we questioned the impact of ferroportin on iron levels and susceptibility towards ferroptosis in MΦ. Towards this, we silenced SLC40A1 using a siRNA approach, resulting in >80% reduction of ferroportin mRNA expression (Fig. 2A). To assess the impact of ferroportin on intracellular iron, we analyzed the cellular labile iron pool (LIP) measuring calcein fluorescence dequenching following the addition of the iron chelator deferiprone by flow cytometry [20]. Ferroportin silencing increased the LIP (Fig. 2B), and caused a small, but significant elevation of RSL3-elicited lipid ROS (Fig. 2C). In addition, the ferroportin knockdown attenuated viability of RSL3-treated MΦ (Fig. 2D).

**Fig. 3:**
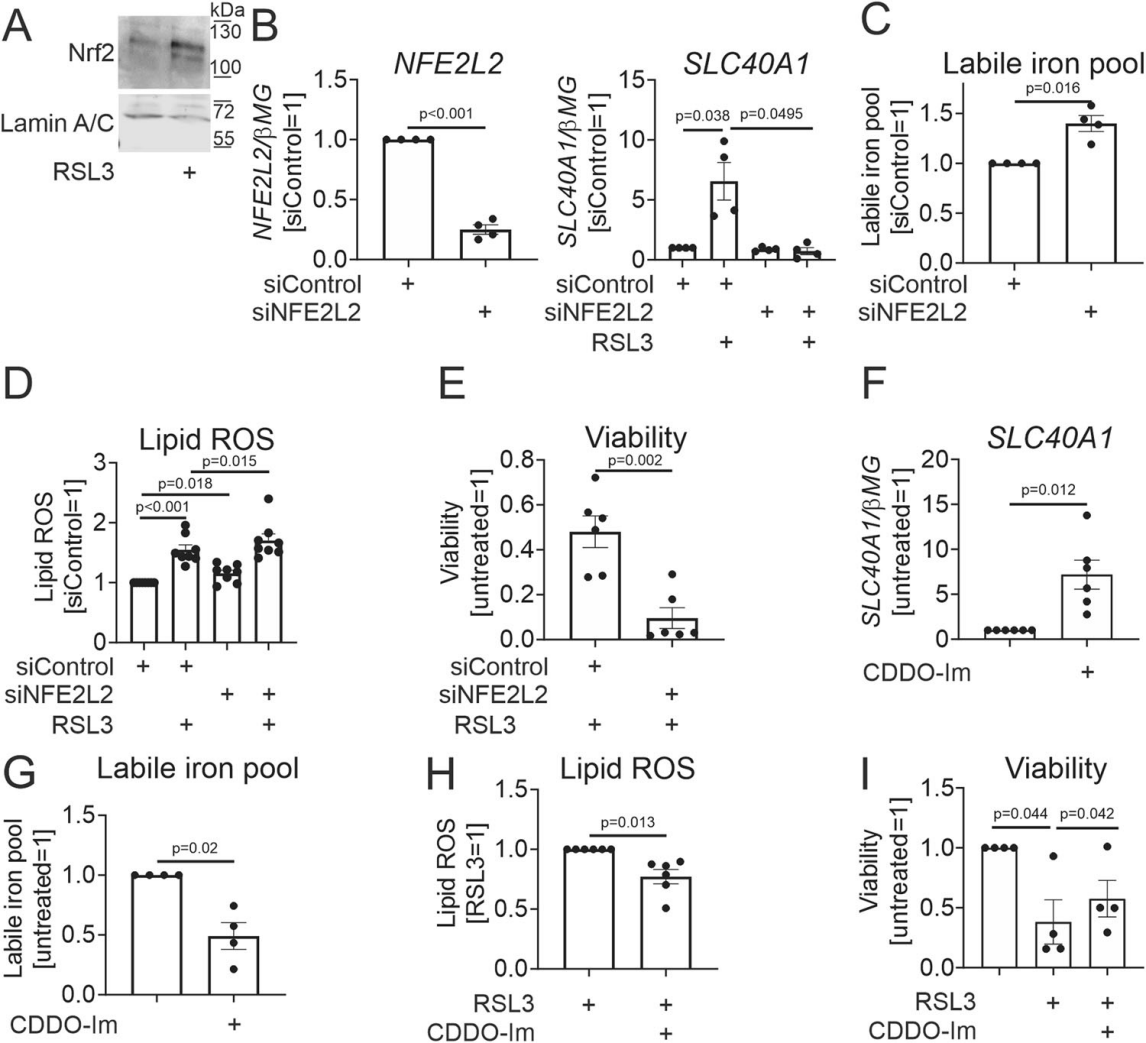
Nrf2 is critical for SLC40A1 induction and cytoprotection in RSL3-treated MΦ. **A** Nrf2 protein expression in nuclear extracts of MΦ exposed to 10 µM RSL3 for 6 h. **B** NFE2L2 and SLC40A1 mRNA expression in MΦ transfected with control or NFE2L2 siRNAs for 96 h prior to treatments with 10 µM RSL3 for 24 h. **C** LIP in MΦ transfected with control or NFE2L2 siRNAs for 96 h. **D**, **E** Lipid ROS (**D**) and viability (**E**) of MΦ transfected with control or NFE2L2 siRNAs for 96 h prior to treatments with 10 µM RSL3 for 6 (**D**) or 24 h (**E**). **F**, **G** SLC40A1 mRNA expression (**F**) and the LIP (**G**) in MΦ exposed to 100 nM CDDO-Im for 6 h (**F**) or 24 h (**G**). **H**, **I** Lipid ROS (**H**) and viability (**I**) of MΦ incubated with 100 nM CDDO-Im for 24 h prior to treatments with 10 µM RSL3 for 3 (**H**) or 24 h (**I**). Data are presented as means ± SEM (*N* ≥ 4).

We then addressed the mechanism of ferroportin induction by RSL3. Ferroportin expression is transcriptionally regulated by the redox-sensitive transcription factor Nrf2 (NFE2L2) [14]. Nrf2 is kept in an inactive state through its interaction with Keap1, which promotes Nrf2 ubiquitination and proteasomal degradation [21]. ROS induce a covalent modification of reactive cysteine residues of Keap1, causing its dissociation from Nrf2 and subsequent nuclear accumulation of Nrf2, where it activates the expression of its target genes, including ferroportin. Indeed, analyzing nuclear extracts of RSL3-treated MΦ, we observed increased levels of Nrf2 protein (Fig. 3A). To question the impact of Nrf2 on ferroportin expression, we silenced the NFE2L2 gene, allowing 75% reduction of Nrf2 mRNA (Fig. 3B). Strikingly, Nrf2 silencing abolished ferroportin induction by RSL3 (Fig. 3B). Consequently, Nrf2 silencing increased the LIP (Fig. 3C). Following RSL3 treatments, Nrf2-silenced MΦ displayed slightly increased lipid ROS (Fig. 3D) and revealed a profound loss of cell viability (Fig. 3E).

To further prove the role of Nrf2, we used a potent pharmacological Nrf2 activator CDDO-imidazole (CDDO-Im) [22]. Treating MΦ with 100 nM CDDO-Im increased ferroportin mRNA (Fig. 3F), and decreased the LIP (Fig. 3G). Furthermore, CDDO-Im pre-treatment attenuated RSL3-triggered lipid ROS (Fig. 3H), and protected RSL3-treated MΦ from ferroptosis (Fig. 3I).

**Fig. 4:**
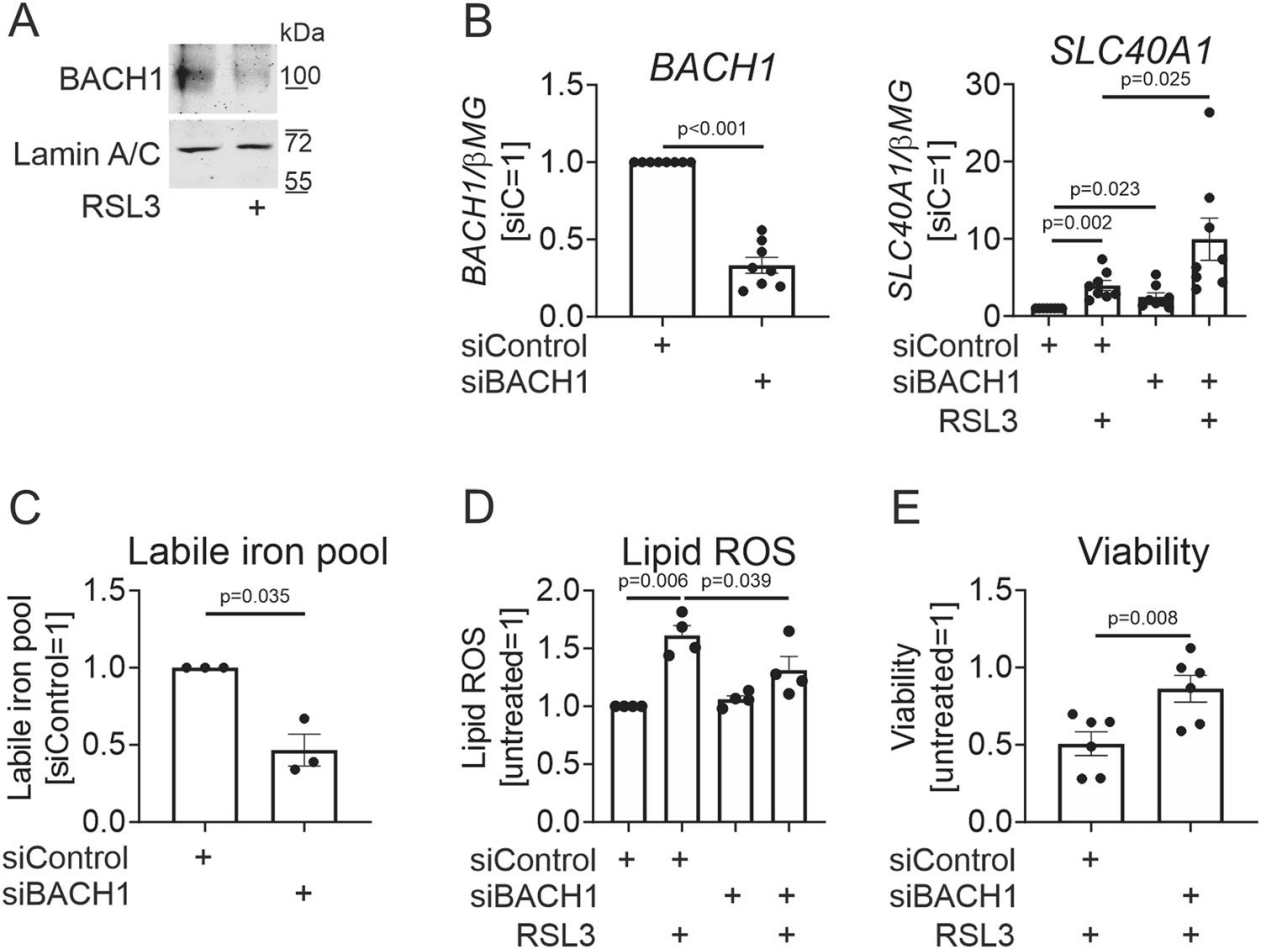
BACH1 silencing increases ferroportin expression and protects against ferroptosis. **A** BACH1 protein expression in nuclear extracts of MΦ exposed to 10 µM RSL3 for 6 h. **B** BACH1 and SLC40A1 mRNA expression in MΦ transfected with control or BACH1 siRNAs for 96 h prior to treatments with 10 µM RSL3 for 24 h. **C** LIP in MΦ transfected with control or BACH1 siRNAs for 96 h. **D**, **E** Lipid ROS (**D**) and viability (**E**) of MΦ transfected with control or BACH1 siRNAs for 96 h prior to treatments with 10 µM RSL3 for 6 (**D**) or 24 h (**E**). Data are presented as means ± SEM (*N* ≥ 3).

In addition to Nrf2, ferroportin expression is regulated by the transcriptional repressor BACH1 [13]. Whereas BACH1 activity is primarily controlled by intracellular heme, it may also undergo redox-dependent nuclear export mediated by oxidative modifications of Cys574 [23]. We observed a drop of BACH1 nuclear protein levels in RSL3-treated MΦ (Fig. 4A). To assess the impact of BACH1 on ferroportin expression, we silenced the BACH1 gene, with a 70% reduction of BACH1 mRNA expression (Fig. 4B). BACH1 knockdown elevated basal as well as RSL3-stimulated ferroportin mRNA expression (Fig. 4B). In addition, BACH1-silenced MΦ exhibited a reduced LIP (Fig. 4C), attenuated lipid ROS (Fig. 4D), and increased cell viability following RSL3 treatments (Fig. 4E). Our data suggest a coordinated response to RSL3, involving upregulation of Nrf2 and downregulation of nuclear BACH1. This includes elevated ferroportin expression, which in turn may protect MΦ against GPX4 inhibition-triggered ferroptosis by reducing the LIP and consequently, attenuating lipid ROS generation.

## Discussion

Human macrophages are, compared to many tumor cells, resistant towards ferroptosis induction, which may in part be due to high ferritin expression [17]. Our findings indicate that ferroportin induction may also contribute to the resistance of MΦ towards GPX4 inhibition. Furthermore, Nrf2 activation or BACH1 suppression may represent an important regulatory mechanism contributing to cytoprotection against ferroptosis, in part by ferroportin upregulation.

MΦ display a different sensitivity towards either GPX4 or system x_c_^–^ inhibition with regard to lipid ROS generation, ferroportin upregulation, and ferroptosis induction. This may suggest that in MΦ, only complete inhibition of GPX4 achieves levels of lipid peroxidation that in turn induce ferroportin expression and trigger ferroptosis. We speculate that an erastin-insensitive pool of reduced glutathione may allow GPX4 or other glutathione peroxidases to prevent lipid peroxidation and ferroptosis. In addition, the increased sensitivity of MΦ towards RSL3 may reflect its interaction with other selenocysteine-containing proteins besides GPX4 [24]. We also noted considerable heterogeneity of responses towards RSL3 between individual blood donors, which may prevented reliable ferroptosis detection in the absence of ferritin silencing in our previous study [17].

Although silencing ferroportin increased the sensitivity of macrophages towards ferroptosis, the effects were of a small magnitude. This suggests that the increase of ferroportin-mediated iron efflux may be an auxiliary mechanism, acting alongside ferritin upregulation [17] to alleviate ferroptosis in RSL3-treated human macrophages.

Mechanistically, our data suggest that either Nrf2 upregulation or BACH1 downregulation may contribute to induce ferroportin expression and thus, cytoprotection after GPX4 inhibition. Our findings concur with the notion that the Nrf2-ferroportin axis controls the LIP in macrophages in response to electrophilic compounds or iron overload [13, 14]. We now added lipid peroxidation-induced Nrf2 activation as a regulatory mechanism. Likely, other transcriptional targets of Nrf2 [25] contribute to cytoprotection, since Nrf2 silencing induces a more profound loss of viability after GPX4 inhibition as compared to ferroportin silencing. These targets may be unrelated in controlling the LIP, as the effects of Nrf2 and ferroportin silencing on the LIP are of similar magnitude.

We also observed that nuclear levels of BACH1 protein were down-regulated after the exposure to RSL3, similar to observations in erastin-treated murine embryonic fibroblasts (MEFs) [26]. This may result from redox modification of BACH1 [23]. BACH1 silencing was clearly associated with ferroportin induction, reduction of the LIP, and cytoprotection, confirming the importance of BACH1 as a negative regulator of ferroportin [13, 27]. Similarly, BACH1 knockout MEFs were resistant to ferroptosis [26], and multiple transcriptional targets of BACH1, including ferroportin, heme oxygenase 1, ferritin, glutamate-cysteine ligase, or SLC7A11, were suggested to contribute to cytoprotective effects of the BACH1 deficiency. Multiple cytoprotective targets are also likely to operate in BACH1-silenced human MΦ.

To conclude, our current and previous findings indicate that multiple mechanisms contribute to human MΦ resistance towards ferroptosis. In particular, we highlight the importance of the Nrf2-BACH1 regulatory system in MΦ cytoprotection. The activation of Nrf2 by ferroptosis-inducing drugs may also suppress pro-inflammatory responses of macrophages, considering known anti-inflammatory properties of Nrf2 [28]. Further research should elucidate how these mechanisms shape the MΦ phenotype in a more complex cellular milieu, such as the tumor microenvironment, to understand the impact of ferroptosis-inducing anti-tumor therapies on tumor-associated macrophages.

## Materials and methods

### Monocyte isolation, differentiation, and treatment

Human peripheral blood mononuclear cells (PBMC) were isolated from commercially available buffy coats from anonymous donors (DRK-Blutspendedienst Baden-Württemberg—Hessen, Institut für Transfusionsmedizin und Immunhämatologie, Frankfurt, Germany) by Pancoll (PAN Biotech, Aidenbach, Germany) density centrifugation. Monocytes were isolated from PBMC by adherence to culture dishes after 1-h incubation in serum-free RPMI1640 medium. Monocytes were differentiated to macrophages in RPMI1640 medium (ThermoFisher Scientific, Waltham, MA, USA) supplemented with 100 U/mL penicillin, 100 μg/mL streptomycin and 3% human serum (DRK-Blutspendedienst Baden-Württemberg—Hessen) for 7 days and cultured thereafter in RPMI 1640 medium containing 10% fetal calf serum. As indicated, cells were treated with RSL3 (#19288), erastin (#17754), liproxstatin-1 (#17730), and CDDO-Imidazole (#31763, all Cayman Chemicals, Ann Arbor, MI, USA). Cell morphology was observed using an Axiovert 40C microscope (Carl Zeiss, Jena, Germany) with an attached Canon EOS 600D camera.

### siRNA transfections

Control siRNA and siRNAs targeting human SLC40A1, NFE2L2, and BACH1 (siGENOME human SMARTpool, Horizon Discovery, Waterbeach, UK) were transfected into macrophages at a final concentration of 50 nM using HiPerFect transfection reagent (Qiagen, Hilden, Germany) according to the manufacturer’s instructions.

### RNA isolation and q-PCR

Total RNA was isolated using TRIzol reagent (Life Technologies, Carlsbad, CA, USA) followed by reverse transcription using Maxima first-strand cDNA synthesis kit (ThermoFisher Scientific). Quantitative real-time PCR (Q-PCR) assays were performed with PowerUp SYBR Green Master Mix (ThermoFisher Scientific) using Quant Studio Real Time PCR System (ThermoFisher Scientific).

### Western blot analysis

For isolation of nuclei, cells were lysed in a nuclear lysis buffer A (20 mM Tris-HCl pH 8.0, 10 mM NaCl, 5 mM EDTA, 0.5% NP-40, 1 mM PMSF, protease inhibitor cocktail) followed by centrifugation at 16,000 × *g* for 20 s. Nuclear pellets were sonicated in lysis buffer B (20 mM Tris-HCl pH 8.0, 400 mM NaCl, 5 mM EDTA, 0.5% NP-40, 1 mM PMSF, protease inhibitor cocktail) followed by centrifugation at 10,000 × *g* for 10 min at 4^ o^C. Supernatants were heat-denatured at 95 °C, separated on SDS-PAGE gels, and transferred onto nitrocellulose membranes. Primary antibodies directed against Nrf2 (#12721, Cell Signalling Technology, Frankfurt, Germany), Bach1 (14018-1-AP, Proteintech, Manchester, UK), and Lamin A/C (#sc-376248, Santa Cruz Biotechnology, Heidelberg, Germany) were used followed by IRDye 680 or IRDye 800-coupled secondary antibodies (LICOR Biosciences, Bad Homburg, Germany). Blots were visualized using LICOR ODYSSEY scanner, and analyzed by Image Studio Digits 5.0 software (LICOR Biosciences).

### Lipid ROS measurements

Cells were treated with RSL3 for 6 h. Afterwards cells were trypsinized, transferred into FACS tubes, and incubated for 10 min at 37 °C with 5 µM BODIPY C11 581/591 (D3861, ThermoFisher Scientific) in Hanks balanced salt solution. Fluorescence in a BB515 channel was recorded using FACSymphony A5 flow cytometer (BD Biosciences, Heidelberg, Germany).

### Measurements of labile iron pool

The labile iron pool was measured based on a published method [20]. Cells were trypsinized, transferred into FACS tubes, and incubated for 1 h at 37 °C with 0.25 µM Calcein-AM (C1430, ThermoFisher Scientific) in the presence or absence of 300 µM iron chelator deferiprone (#20387, Cayman Chemicals). Fluorescence in a BB515 channel was recorded using FACSymphony A5 flow cytometer (BD Biosciences). The labile iron pool was defined as a difference in Calcein median fluorescence intensity of samples labeled with and without deferiprone.

### CellTiter-Blue® viability assay

Cells were cultured in 48-well plates. Viability was analyzed using CellTiter-Blue® Cell Viability Assay (Promega, Walldorf, Germany) according to manufacturer’s instructions.

### Lactate dehydrogenase release assay

Cells were treated with RSL3 for 24 h. Lactate dehydrogenase (LDH) activity in culture medium and in cell lysates was measured using the LDH Cytotoxicity Assay Kit (88953, ThermoFisher Scientific) according to manufacturer’s instructions.

### Statistical analysis

Statistical analysis was performed using GraphPad Prism 8.0 (GraphPad, San Diego, CA, USA). The sample size for each experiment was estimated empirically, according to literature using similar experimental systems. Normal distribution was assessed using Shapiro–Wilk test. Data were analyzed using Student paired, two-tailed *t*-test, one sample *t*-test, or by one-way ANOVA with Bonferoni multiple comparisons. Graphical data are presented as means ± SEM with dots indicating individual biological replicates.

## Supplementary information

Original Data File

## Data Availability

The corresponding author will provide the original data used to support the findings of this study upon reasonable request.
