# Supplementary figures and images for: Interplay of Nrf2 and BACH1 in inducing ferroportin expression and enhancing resistance of human macrophages towards ferroptosis
Source: Cell Death Discov. 2022 Jul 19;8:327. doi: 10.1038/s41420-022-01117-y (PMC9296510; doi:10.1038/s41420-022-01117-y)

Fig 3A

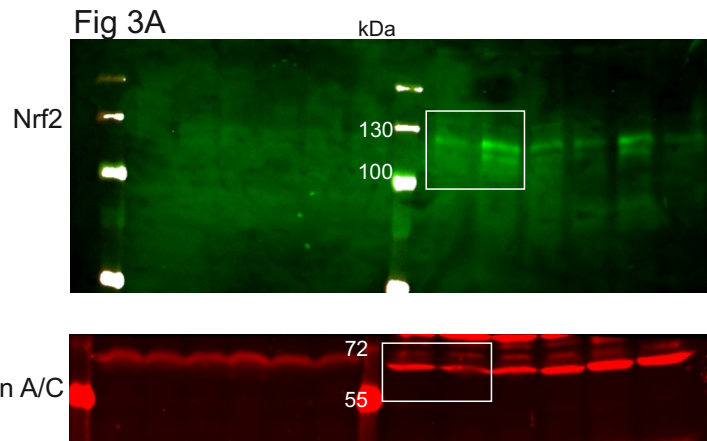

Fig 4A

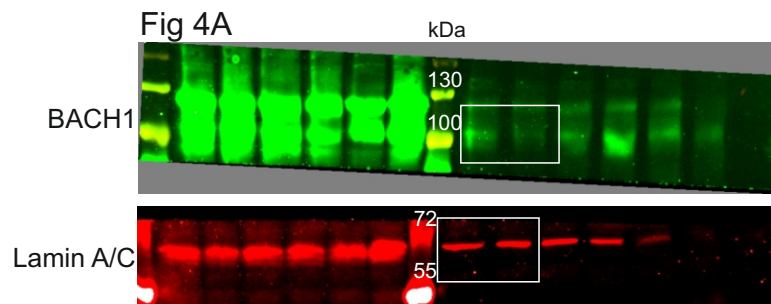

Supplement: Supplementary file 1 — Original Data File [file 41420_2022_1117_MOESM1_ESM.pdf]
